# What are the acceptances and associated influences of hospice care in Mainland China? A national cross-sectional study

**DOI:** 10.3389/fpubh.2022.985218

**Published:** 2022-09-23

**Authors:** Xinyue Zhang, Xun Zhang, Yiqi Li, Tianle Chen, Lixuen Siow, Xinxin Ye, Yinlin Wang, Yujia Wang, Wai-Kit Ming, Xinying Sun, Ze Xiang, Yibo Wu, Jian Wu

**Affiliations:** ^1^Key Laboratory of Oral Biomedical Research of Zhejiang Province, Stomatology Hospital, School of Stomatology, Zhejiang University of Medicine, Zhejiang Provincial Clinical Research Center for Oral Diseases, Cancer Center of Zhejiang University, Hangzhou, China; ^2^Department of Surgical Oncology, Sir Run Run Shaw Hospital, Zhejiang University, Hangzhou, China; ^3^Zhejiang University School of Medicine, Hangzhou, China; ^4^Chu Kochen Honors College of Zhejiang University, Zhejiang University, Hangzhou, China; ^5^School of Public Health, Zhejiang University School of Medicine, Zhejiang University, Hangzhou, China; ^6^College of Humanities and Social Sciences, Harbin Medical University, Harbin, China; ^7^Department of Infectious Diseases and Public Health, Jockey Club College of Veterinary Medicine and Life Sciences, City University of Hong Kong, Kowloon, Hong Kong SAR, China; ^8^Department of Social Medicine and Health Education, School of Public Health, Peking University, Beijing, China; ^9^Department of Laboratory Medicine, The Affiliated Suzhou Hospital of Nanjing Medical University, Suzhou Municipal Hospital, Gusu School, Nanjing Medical University, Suzhou, China

**Keywords:** hospice care, personal intention, affecting factors, cross-sectional study, China, public education

## Abstract

**Background:**

China ranks 53^rd^ out of 81 countries in the Quality of Death Index for 2021. Although hospice care demand is increasing, the progress remains slow. It is of great significance to explore the acceptances and associated influencing factors of hospice care.

**Methods:**

A cross-sectional survey by quota sampling was conducted in China from July 10^th^ to September 15^th^, 2021. We collected demographic data and hospice care acceptance. A stepwise linear regression analysis was used.

**Results:**

This survey contained 11,031 valid questionnaire results to investigate the hospice care acceptance. It was found that individuals with undergraduate or above (β = 0.04), more properties [2 (β = 0.02), 3 (β = 0.01)], and higher reimbursement types of medical insurance [employee health insurance and commercial health (β = 0.03), government insurance (β = 0.04)] had higher hospice acceptance willingness, while males (β = −0.02) were less willing to accept than females. Psychological conditions [mild anxiety (β = 0.03), moderate anxiety (β = 0.01), moderate stress (β = 0.05), and severe stress (β = 0.06)] also played an important role. The Self-Management Scale (SHMS) (β = 0.12), EuroQol Five Dimensions Questionnaire (EQ-5D) (β = 0.05), EuroQol Visual Analog Scale (EQ-VAS) (β = 0.21), Short-Form Family Health Scale (FHS-SF) (β = 0.12), higher scores of the Short-Form Health Literacy Instrument (HLS-SF12) (β = 0.16), and Perceived Social Support Scale (PSSS) (β = 0.10) also contributed. Gender subgroup showed that in the male group, age, highest educational level, marital status, number of properties, whether having children, psychological conditions, the SHMS, EQ-5D, EQ-VAS, HLS-SF12, and PSSS showed significant difference. Urban and rural subgroups showed that age, highest educational level, number of properties, whether having chronic disease or psychological conditions, the SHMS, EQ-VAS, HLS-SF12, and PSSS were contributing factors in rural areas.

**Conclusion:**

The average score of acceptance of hospice care was 65.02 points. Gender, house, anxiety, pressure, social support, and health literacy were the main influencing factors on residents' attitudes.

## Background

In 2021, a study on the Quality of Death Index showed that China ranked 53^rd^ out of 81 countries, indicating that there is still much room for improvement in the quality of death in China (1). China is a large developing country with a population of more than 1.4 billion, accounting for one-fifth of the world (2). In recent years, the growing elderly population has led to a demographic change (3). The growing trend of population aging, especially later in life, magnifies the need for hospice services.

Hospice care services can improve quality of life, save costs, and shorten hospital stays. Generally, high-income countries have higher access to hospice care but are also affected by geographical differences, such as the England and Canada (4). Hospice care is generally lower in developing countries and is more influenced by economics, education, and national policies (3). In China, the aging population has led to greater demand for hospice care services (5). However, the development of hospice care progresses very slowly and the supply of hospice care is limited in Mainland China, while hospice care services are widely provided in Western countries. Another obstruction is that death has long been a taboo in traditional Chinese culture, which may result in the concept that death is a failure caused by medical treatment (6). In a multicenter cross-sectional study, only 10.5% of 1,084 patients preferred a hospice facility (7). Besides, the economic and cultural conditions vary a lot in different parts of China, which may also affect the acceptance of hospice care. A previous study reported that attitudes toward hospice care are crucial to end-of-life decisions and that different individual characteristics influence preferences for hospice care (8). Nevertheless, there is no nationwide sample of the Chinese population to explore the factors associated with hospice care.

Hence, our study investigates people's attitudes toward palliative and hospice care and analyzes the associations between attitudes and potential explanatory variables based on the nationwide population sample in China. Given what we know, this study is the first to discuss the general intention of hospice care. This is likely to contribute to the development of hospice care by promoting global access to hospice care and quality of death.

## Methods

### Ethics statements

This study scheme was approved by the Institutional Review Committee of Jinan University, Guangzhou, China (JNUKY-2021-018). The questionnaire survey (Chinese Family Health Index survey, 2021) for the study was formally sponsored by China Family Newspaper and designed by Peking University, Jinan University, and Beijing Jiaotong University. All respondents volunteered to participate in the survey.

### Survey design

The survey was carried out from July 10^th^, 2021 to September 15^th^, 2021, which involved 22 provinces, 5 autonomous regions, and 4 municipalities all over China's mainland. There were 120 cities, including the capital city, and 2–6 other prefecture-level cities of each province and autonomous region chosen by random number table method. The investigators or investigator teams were openly recruited in each chosen city to obtain the samples by quota sampling of the citizens, whose gender, age, and urban and rural distribution are in line with the demographic characteristics of the 7th National Census in 2021 (1–3). Each city was assigned at least 1 investigator (or team) by using the wenjuanxing platform (https://www.wjx.cn/, a professional online questionnaire survey platform). Moreover, we conduct a rigorous training for the investigators we recruited for the questionnaire survey, which included the standardized process and emergency measures to cope with the survey. The investigators imported the survey questionnaire into the wenjuanxing system and created a link. Then they only need to share the link with the respondents through the online platform. Each investigator needs to collect 30–90 questionnaires (100–200 for each team) according to the set requirements. After giving informed consent to the survey, these respondents could answer the questionnaire and had the right to abandon it during the survey, and all the respondents remained anonymous.

Specific quota sampling criteria (every 100 samples) include: (1) Age criteria: under 18: 8 ± 5 (samples); between 19–24: 12 ± 5; between 25–30: 12 ± 5; between 31–40: 16 ± 5; between 41–50: 18 ± 5; between 51–60: 18 ± 5; between 51–60: 18 ± 5; between 61–70: 10 ± 5; over 71: 18 ± 5. (2) Gender criteria: the proportion between male and female is close to 1:1. (3) Urban and rural distribution criteria: the proportion between urban and rural samples is close to 3:2.

Inclusion criteria for this study include: (1) Age ≥ 12 years. (2) The nationality of the People's Republic of China. (3) China's permanent resident population (annual travel time ≤ 1 month). (4) Participate in the study voluntarily and fill in the informed consent form. (5) Participants can complete the network questionnaire survey by themselves or with the help of investigators. (6) Participants can understand the meaning of each item in the questionnaire. The reasons why we chose teenagers aged above 12 for our study are as follows. Firstly, we found that teenagers as well as adults have already accepted death education due to the improvement in education quality and social milieu (9, 10). Most teenagers aged above 12 have the conditions and circumstances to form their own outlooks of life and death, so they may also have mature ideas about hospice care. Secondly, those teenagers are fully capable of reading the questionnaire and answering it online with their smartphone (if they have). Taking these two points into consideration, it was believed that those teenagers would finish the survey rationally. Exclusion criteria include: (1) Those with mental disorders or insanity. (2) Those involved in other similar research projects. (3) Those unwilling to cooperate.

A total of 1,1709 questionnaires were collected and we screened 1,1031 valid questionnaires, with the 94.2% effective rate. In the process of the survey and analysis, we held 3 expert meetings to discuss and modify our research approach, which was advised by experts in different fields. Also, the final structure and data of the survey was rigorously screened and audited by those experts, and proved to be valid and meaningful.

### Research instrument

Hospice care is a particular therapeutic method that provides physical, psychological and humanistic care for terminal and elderly people at their dying time. It may relieve their pain and discomfort and improve the quality of their lives, leading them to a peaceful and serene death. To discover more valuable information, the study focused on the relevance between residents' intentions for hospice care (according to what the investigators interpreted for the respondents) and their health status. After consulting professionals, we used a visual analog scale (VAS) scoring from 0 to 100 to assess their intention toward hospice care, with a higher score representing a stronger willingness to accept hospice care. Besides, based on the previous literature and information from other studies (11), the study mainly analyzed some sociodemographic characteristics (gender, age, house properties and so on) and health indicators (self-management ability, life quality, social support degree and so on) as the representative health metrics to assess their health status.

In this study, in addition to commonly used demographic and sociological characteristics, we added some health-related scales to measure the correlation between the respondents' multi-faceted health status and their willingness to accept hospice care. The Self-Management Scale (SHMS) can reflect the respondents' health management ability, and show the importance of the respondents to health. A previous study showed that the EuroQol Five Dimensions Questionnaire (EQ-5D), a simple and effective quality-of-life assessment tool, can serve as a useful predictor of survival in advanced cancer patients receiving hospice care (12). And for EQ-VAS, it can reflect the respondents' perception of their own health status and have an impact on the respondents' attitudes toward health. Social support also plays an important role in end-of-life care (13), and the Perceived Social Support Scale (PSSS) will reflect the support that the respondents feel. Both the Short-Form Health Literacy Questionnaire (HLS-SF) and Short-Form Family Health Scale (FHS-SF) can reflect the health literacy of respondents and their home environment. It has been confirmed that health literacy is related to respondents' knowledge, attitudes and decisions about end-of-life care (14). Besides, a scale measuring the psychological state of the respondents was also added to observe the influence of psychological state on the willingness to accept hospice care.

SHMS was used to measure self-management ability. The SHMS is made up of 3 sub-scales and they assess exercise frequency, cognitive symptom management, and communication ability with doctors. According to their personal conditions, respondents choose the corresponding options and those options would be rated from 0 to 5 (Likert-type). Then all the items would be summed up for a total score between 6 and 75, with higher scores indicating better self-management ability. The Cronbach's alpha of the SHMS was 0.79 (15).

EQ-5D was used to measure life quality and a sense of self-health. For this study, we used the five-level version (EQ-5D-5L). There are 5 items in the questionnaire that assess the action, self-care, daily activities, pain or discomfort and depression of respondents. Items would also be rated from 1 to 5 (Likert-type) and summed up. The total score is between 5 and 25 with higher scores indicating lower life quality. Additionally, EQ-VAS is also used to assess the sense of self-health. The VAS scales range from 0 to 100, with a higher score indicating a better sense of self-health. The respondents input a number that reflects their health conditions from their own perspective. The Cronbach's alpha of the EQ-5D-5L was 0.87 (16).

A 3-item self-made scale was used to measure the sense of self-pressure. The scale includes the following items: (1) How would you evaluate your ability to cope with stress? (2) How would you evaluate the stress level in your life (at home and at work) over the last two weeks? (3) How would you evaluate the stress level in your life (at home and at work) over the last year? For each item, respondents inputted a number from 1 to 6 which indicated their stress levels on both family and work. The summed scale has a total score of between 3 and 18, with a higher score indicating more significant stress perception. The total score was used to classify the stress status of the respondents: a score of 3–6 means mild pressure, 7–15 means moderate pressure, and 16–18 means severe pressure.

PSSS was used to assess the degree of social support. PSSS assesses the perception of emotional support from social relationships through 12 items. Items are rated from 0 to 6 (Likert-type), based on the options from “very strongly disagree” to “very strongly agree.” The summed items have a total score between 0 and 72, with a higher score indicating a higher degree of the perception of social support. The Cronbach's alpha of the PSSS was 0.91 (17).

HLS-SF was used to measure health literacy. For the study, we used the 12-item version (HLS-SF12). HLS-SF12 assesses the ability to find, understand, assess and apply health-related information. Items are rated from 1 to 4 (Likert-type), based on the options from “very hard” to “very easy”. The summed items have a total score of between 0 and 72, with a higher score indicating a better health literacy. The Cronbach's alpha of the HLS-SF12 was 0.87 (18).

FHS-SF was used to measure family health degree. FHS-SF assesses 4 influencing factors including social and emotional health processes, health lifestyle, health resources, and external social support of a family through 10 items. The total score is summed up by rating each item from 1 to 5. The final score is between 10 and 50, with a higher score indicating a higher family health degree (19).

The Generalized Anxiety Disorder-7 (GAD-7) was used to measure anxiety status. 7 items are included to assess the frequency of anxiety symptoms by scoring each item from 0 to 3, and the total score ranges from 0 to 21. According to the reference standard, a score of 0-4 reflects no anxiety, 5–9 reflects mild, 10–14 reflects moderate, and 15–21 reflects severe symptoms. The Cronbach's alpha of the GAD-7 was 0.87 (20).

The Patient Health Questionnaire (PHQ-9) used nine items scored on their frequency from “not at all” to “nearly every day” resulting in a score between 0 and 27 (higher scores representing greater depressive symptoms) to assess depressive disorders. The Cronbach's alpha of the PHQ-9 was 0.84 (21).

### Statistical methods

We used SPSS™ for Windows, Version 26.0 (SPSS, Inc., Chicago, IL, USA) for our data analysis. The descriptive analysis includes the mean value, standard deviation (SD) of continuous variables, and the *p*-value of each variable. The ANOVA analysis and t-test were used to compare differential factors for the scores of hospice care intention. Furthermore, a stepwise regression analysis was used to assess those differential variables associated with the intention of hospice care (Inclusion and exclusion criteria were: *P* = 0.05 and *P* = 0.01).

## Results

### Basic characteristics of the 1,1031 respondents

**Figure 1:**
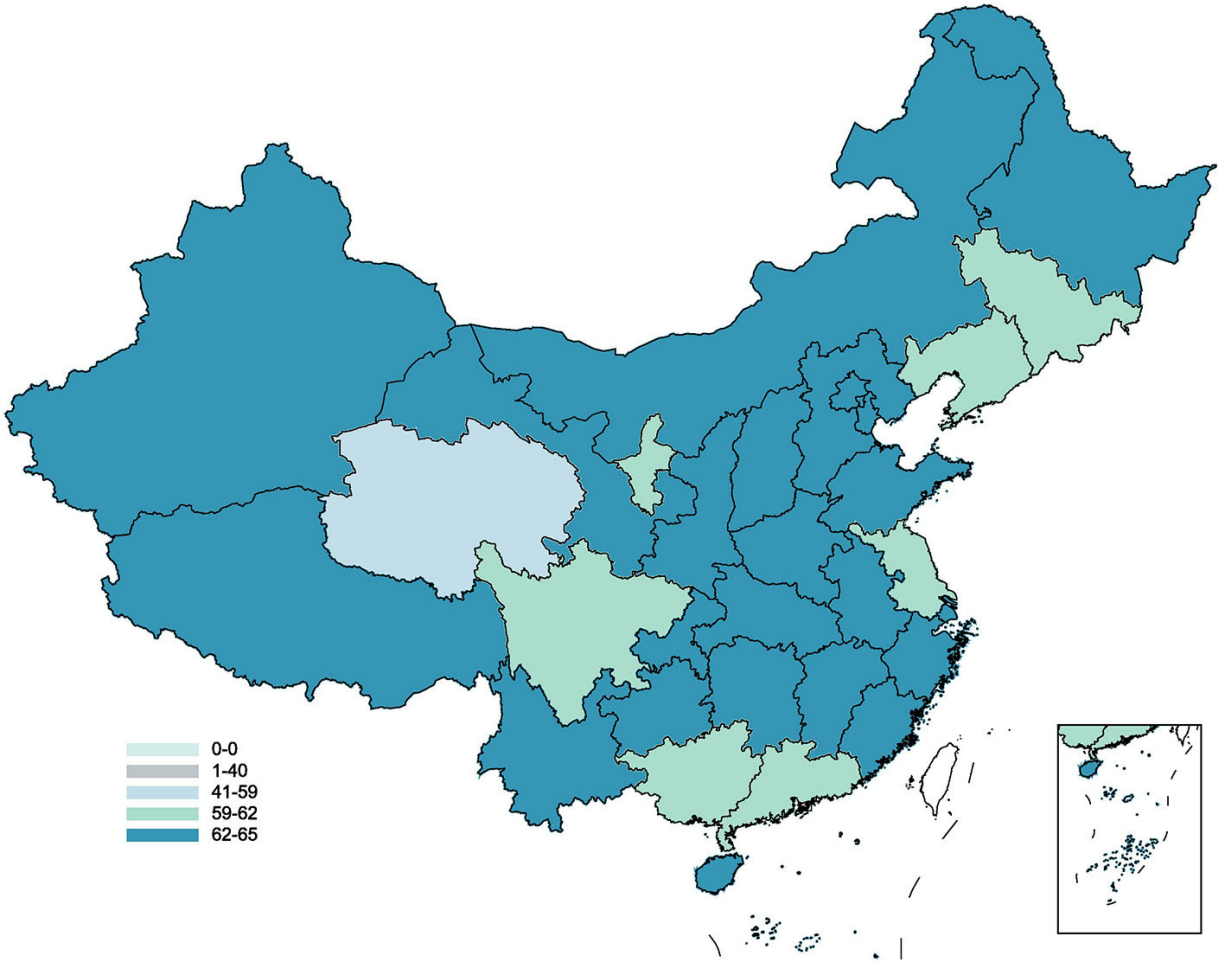
The distribution of acceptance of hospice care in China.

In this survey, a total of 1,1031 valid questionnaire survey results from 22 provinces, 5 autonomous regions, and 4 municipalities all over China's mainland (Figure 1) were shown. 50.87, 25.85, and 23.29% were from the east, central, and western China, respectively. Among the respondents, 54.27% were female, 72.60% lived in urban areas, 42.29% were under the age of 30, 45.71% were undergraduate or above, and 48.52% had resident medical insurance.

**Figure 2:**
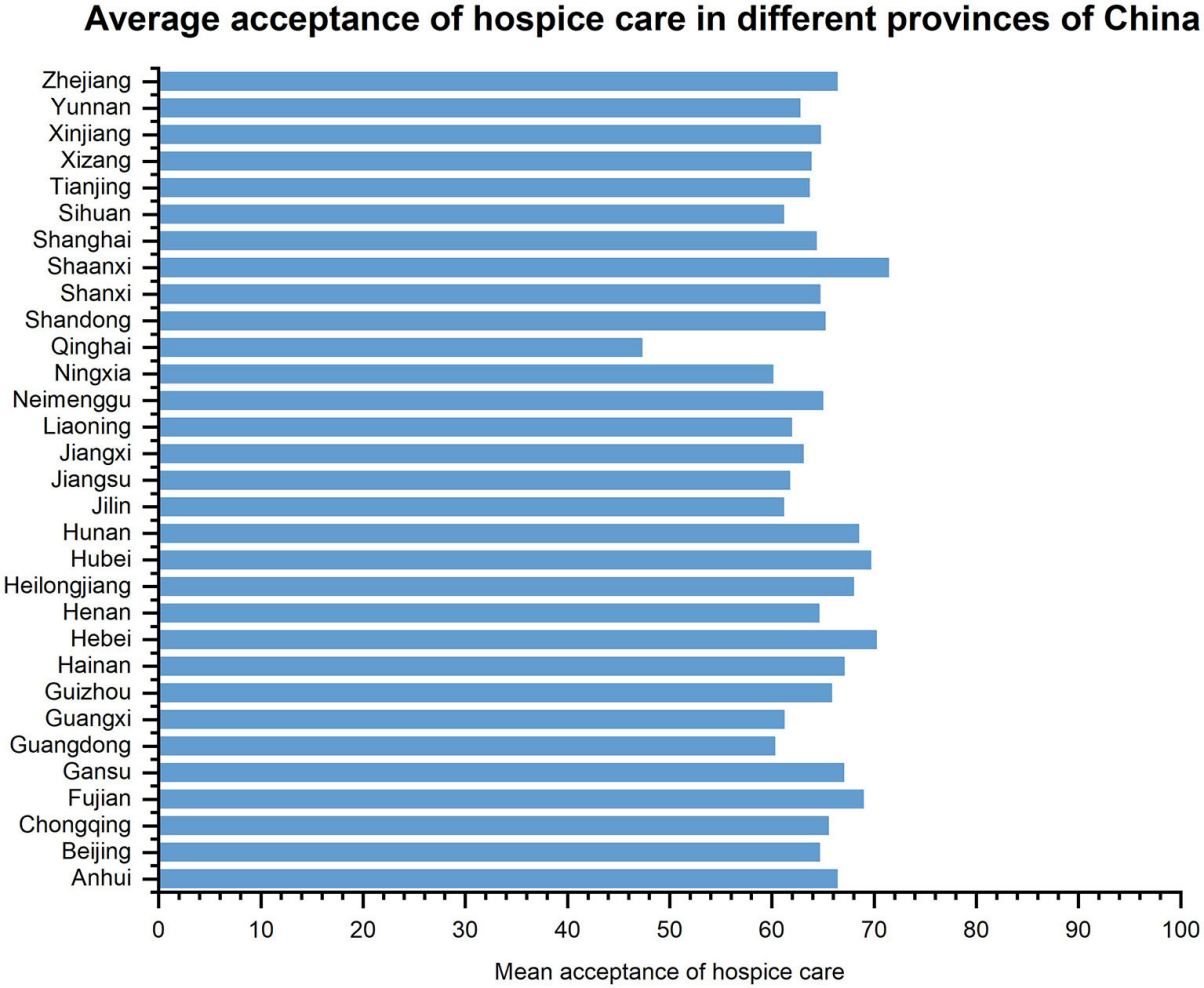
Average acceptance of hospice care in different provinces of China.

**Figure 3:**
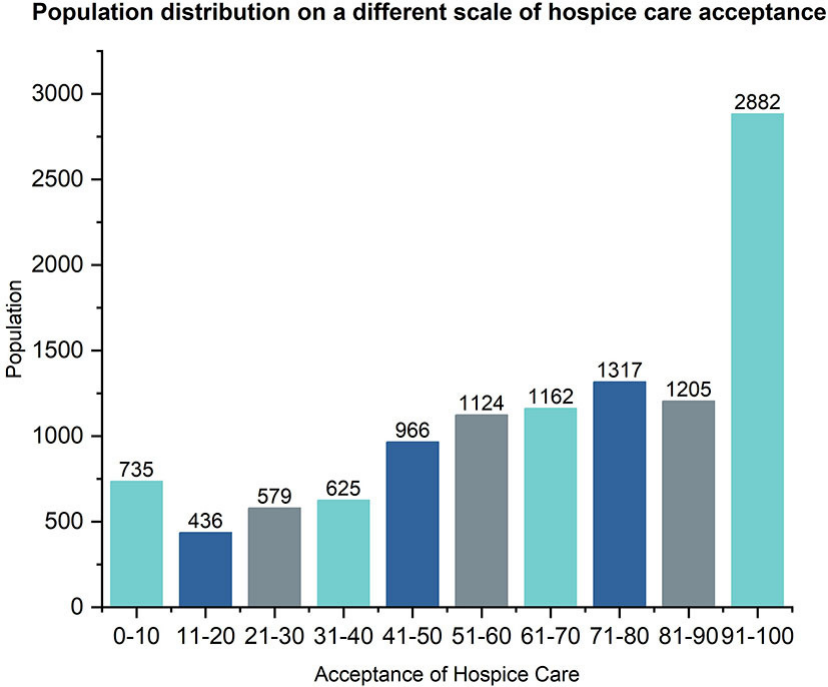
Population distribution on a different scale of hospice care acceptance.

The average score of the PSSS of the survey target group was 48.22 ± 13.03 points, the short-form of the FHS-SF was 37.99 ± 6.64 points, the EQ-5D was 5.84 ± 1.82 points, the HLS-SF12 was 36.70 ± 6.04 points, and the SHMS was 26.49 ± 9.79 points. The average score for willingness to accept hospice care was 65.02 ± 29.82 points. The average willingness to receive hospice care varied across regions (Figure 2). Most of the respondents had a high acceptance rating (Figure 3).

### Acceptance and associated influences of hospice care

To study the factors related to the acceptance of hospice care, we first conducted a chi-square univariate analysis of the variables, and the results showed that the differences in gender, permanent residence, age, highest education level, marital status, number of houses, occupational status, medical insurance type, whether having children, debt, or history of smoking, number of medications taken daily, frequency of drinking, anxiety state, stress state, and depression state were all statistically significant (Table 1). These variables and scales were further analyzed by multiple stepwise linear regression (Table 2). The results showed that among the survey respondents, males (β = −0.02) were less willing to accept than females. Those with undergraduate or above (β = 0.04), more properties [2 (β = 0.02), ≥3 (β = 0.01)], and higher reimbursement types of medical insurance [employee health insurance and commercial health (β = 0.03), government insurance (β = 0.04)] had a higher willingness to accept. The results showed that the psychological state of the respondents also had a great influence on the acceptance level. Compared with the respondents with no anxiety, those with mild anxiety (β = 0.03) and moderate anxiety (β = 0.01) had higher acceptance willingness, while there existed no significant difference in severe anxiety. Compared with respondents with mild stress, those with moderate stress (β = 0.05) and severe stress (β = 0.06) also showed better willingness to accept. SHMS (β = 0.12), EQ-5D (β = 0.05), EQ-VAS (β = 0.21), FHS-SF (β = 0.12), HLS-SF12 (β = 0.16), and PSSS (β = 0.10) also contributed to the results.

**Table 1:** Descriptive statistics of hospice acceptance among study subjects.

**Variate**	**Number**	**Proportion**	**Mean**	***SD***	***F***	***P***
Total	11031	100.00%	65.02	29.82	NA	NA
Gender						
Male	5033	45.63%	63.62	29.97	20.26	<0.001
Female	5998	54.37%	66.19	29.65		
Place of residence						
Urban	8008	72.60%	65.81	29.67	20.73	<0.001
Rural	3023	27.40%	62.91	30.12		
Age group						
<30	4665	42.29%	66.15	29.85	10.89	<0.001
30–44	3001	27.21%	65.76	28.61		
45–59	2218	20.11%	63.61	30.09		
≥60	1147	10.40%	61.17	31.86		
Region						
Eastern China	5611	50.87%	64.92	30.21	1.46	0.232
Central China	2851	25.85%	65.76	29.38		
Western China	2569	23.29%	64.40	29.44		
Highest educational level						
Junior high or below	2566	23.26%	60.92	31.36	51.10	<0.001
Senior high or specialty	3423	31.03%	63.83	29.26		
Undergraduate or above	5042	45.71%	67.91	29.09		
Marital status						
Unmarried	4805	43.56%	66.19	29.87	9.38	0.002
Married	6226	56.44%	64.25	29.64		
Number of propertes owned						
0	1083	9.82%	62.82	30.05	14.15	<0.001
1	6598	59.81%	64.02	29.67		
2	2440	22.12%	67.22	29.86		
≥3	910	8.25%	68.94	29.85		
Occupational status						
Student	3314	30.04%	66.82	29.74	18.52	<0.001
Incumbency	4637	42.04%	65.88	29.25		
Retire	884	8.01%	60.39	31.89		
No fixed occupation	2196	19.91%	62.32	29.91		
Occupation type						
Personnel of government agencies	1050	9.52%	67.50	29.52	7.16	<0.001
Professional and technical personnel	850	7.71%	65.71	29.93		
Medical staff	501	4.54%	70.09	29.70		
Labor service personnel	1729	15.67%	63.44	28.89		
Other	6901	62.56%	64.58	30.04		
Type of medical insurance						
Self-pay	2299	20.84%	64.05	29.57	3.22	0.012
Resident insurance	5352	48.52%	64.54	29.83		
Employee health insurance	2937	26.62%	66.17	30.07		
Commercial health insurance	237	2.15%	67.68	28.17		
Government insruance	206	1.87%	68.50	30.15		
Whether having children						
YES	5969	54.11%	63.78	29.95	13.31	<0.001
NO	5062	45.89%	66.47	29.60		
Whether having debts (including car loans, mortgages)						
YES	4251	38.54%	65.87	29.48	5.62	0.018
NO	6780	61.46%	64.48	30.02		
Whether having chronic disease						
NO	8984	81.44%	65.25	29.79	3.09	0.079
YES	2047	18.56%	63.97	29.93		
Whether having a history of smoking						
YES	2186	19.82%	63.41	30.44	7.94	0.005
NO	8845	80.18%	65.41	29.65		
Number of medications taken daily						
0	8996	81.55%	65.40	29.75	4.13	0.016
1–2	1540	13.96%	63.55	29.88		
≥3	495	4.49%	62.67	30.69		
Anxiety						
No anxiety	6170	55.93%	63.67	31.25	12.49	<0.001
Mild anxiety	3364	30.50%	66.23	28.09		
Moderate anxiety	1198	10.86%	66.95	26.35		
Severe anxiety	299	2.71%	71.50	29.89		
Pressure						
Mild pressure	2719	24.65%	63	34	82.15	<0.001
Moderate pressure	7653	69.38%	65	28		
Severe pressure	659	5.97%	79	27		
Depression						
No depression	5031	45.61%	63.57	31.46	8.00	<0.001
Mild depression	3801	34.46%	65.78	28.67		
Moderate depression	1148	10.41%	65.54	27.85		
Moderate to severe depression	803	7.28%	67.81	26.57		
Severe depression	248	2.25%	71.07	30.14		

**Table 2:** Stepwise regression analysis of factors related to acceptance of hospice care.

**Variables**	**Unstandardized coefficients**		**Standardized coefficients**	***t***	***P***	**95%** ***CI***	
	***B***	***SE***	***Beta***			**Lower limit**	**Upper limit**
Gender (Ref: Female)							
Male	−2.44	0.56	−0.02	−4.39	<0.001	−3.53	−1.35
Highest educational level (Ref: Junior high or below)							
Undergraduate or above	4.21	0.74	0.04	5.65	<0.001	2.75	5.67
Number of propertes owned (Ref: 0)							
2	2.98	1.07	0.02	2.78	0.005	0.88	5.08
≥3	3.28	1.32	0.01	2.49	0.013	0.70	5.86
Type of medical insurance (Ref: Self–pay and resident insurance)							
Employee health insurance and commercial health insurance	3.79	1.76	0.03	2.15	0.031	0.34	7.23
Government insruance	3.65	1.68	0.04	2.17	0.030	0.35	6.94
Anxiety (Ref: No anxiety)							
Mild anxiety	3.66	0.67	0.03	5.47	<0.001	2.35	4.98
Moderate anxiety	2.94	1.05	0.01	2.80	0.005	0.88	4.99
Pressure (Ref: Mild pressure)							
Moderate pressure	4.42	0.68	0.05	6.48	<0.001	3.08	5.76
Severe pressure	17.87	1.34	0.06	13.33	<0.001	15.25	20.50
SHMS	0.30	0.03	0.12	9.51	<0.001	0.24	0.36
EQ−5D	0.63	0.15	0.05	4.15	<0.001	0.33	0.93
EQ–VAS	0.18	0.02	0.21	10.63	<0.001	0.14	0.21
FHS–SF	0.21	0.05	0.12	3.98	<0.001	0.11	0.32
HLS–SF12	0.31	0.05	0.16	5.97	<0.001	0.21	0.41
PSSS	0.14	0.03	0.10	5.14	<0.001	0.09	0.20

### Subgroup analysis of hospice care acceptance of different genders

**Figure 4:**
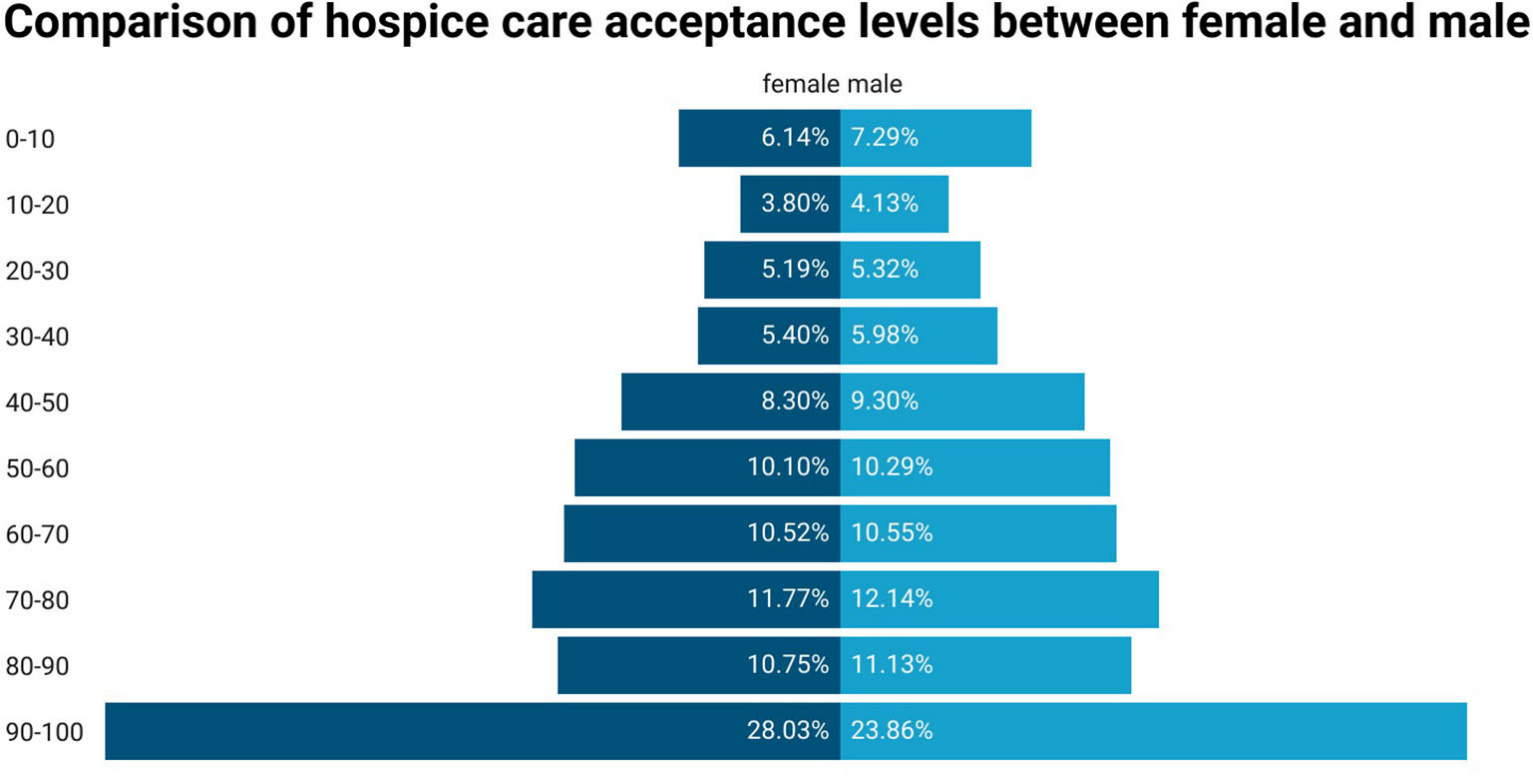
Comparison of hospice care acceptance levels between female and male.

In view of the statistical differences in gender (Figure 4), we further performed a stepwise regression analysis of gender subgroups (Table 3). In the female group, similar to the overall population, the highest educational level [undergraduate or above (β = 0.05)], psychological status [mild anxiety (β = 0.03), moderate anxiety (β = 0.02), moderate stress (β = 0.04) and severe stress (β = 0.05)], and the six scales [SHMS (β = 0.10), EQ-5D (β = 0.06), EQ-VAS (β = 0.18), FHS-SF (β = 0.15), HLS-SF12 (β = 0.23), and PSSS (β = 0.09)] also revealed correlations.

**Table 3:** Hierarchical stepwise regression analysis of predictors of hospice acceptance by gender.

**Variables**	**Unstandardized coefficients**		**Standardized coefficients**	***t***	***P***	**95%** ***CI***	
	***B***	***SE***	***Beta***			**Lower limit**	**Upper limit**
**Female**							
Highest educational level (Ref: Junior high or below)							
Undergraduate or above	5.21	0.98	0.05	5.32	<0.001	3.29	7.14
Anxiety (Ref: No anxiety)							
Mild anxiety	4.33	0.89	0.03	4.86	<0.001	2.58	6.07
Moderate anxiety	3.66	1.45	0.02	2.53	0.012	0.82	6.51
Pressure (Ref: Mild pressure)							
Moderate pressure	3.81	0.92	0.04	4.15	<0.001	2.01	5.61
Severe pressure	14.95	1.89	0.05	7.91	<0.001	11.24	18.66
SHMS	0.27	0.04	0.10	6.09	<0.001	0.18	0.35
EQ−5D	0.75	0.20	0.06	3.68	<0.001	0.35	1.15
EQ–VAS	0.16	0.02	0.18	7.17	<0.001	0.11	0.20
FHS–SF	0.28	0.07	0.15	3.97	<0.001	0.14	0.42
HLS–SF12	0.45	0.07	0.23	6.49	<0.001	0.31	0.58
PSSS	0.14	0.04	0.09	3.52	<0.001	0.06	0.21
**Male**							
Age group (Ref: <30)							
≥60	5.60	1.88	0.03	2.98	0.003	1.92	9.29
Highest educational level (Ref: Junior high or below)							
Undergraduate or above	3.64	1.17	0.03	3.11	0.002	1.35	5.94
Marital status (Ref: Unmarried)							
Married	3.42	1.35	0.08	2.54	0.011	0.78	6.06
Number of propertes owned (Ref: 0)							
≥3	4.84	1.91	0.02	2.54	0.011	1.10	8.59
Whether having children (Ref: No)							
YES	−4.20	1.57	−0.04	−2.67	0.008	−7.28	−1.12
Anxiety (Ref: No anxiety)							
Mild anxiety	2.89	1.01	0.02	2.86	0.004	0.91	4.87
Moderate anxiety	1.53	1.50	0.01	1.02	0.308	−1.41	4.46
Severe anxiety	−6.58	2.69	−0.02	−2.45	0.015	−11.86	−1.30
Pressure (Ref: Mild pressure)							
Moderate pressure	5.40	0.99	0.06	5.45	<0.001	3.46	7.34
Severe pressure	20.39	1.90	0.08	10.74	<0.001	16.67	24.12
SHMS	0.35	0.05	0.15	7.52	<0.001	0.26	0.44
EQ−5D	0.51	0.21	0.04	2.42	0.016	0.10	0.92
EQ–VAS	0.22	0.02	0.26	9.42	<0.001	0.17	0.27
HLS–SF12	0.23	0.07	0.12	3.18	0.001	0.09	0.37
PSSS	0.19	0.04	0.14	5.48	<0.001	0.12	0.26

In the male group, except for the highest educational level [undergraduate or above (β = 0.03)], psychological state [mild anxiety (β = 0.02), moderate anxiety (β = 0.01), severe anxiety (β = −0.02), moderate stress (β = 0.06) and severe stress (β = 0.08)] and the five scales [SHMS (β = 0.15), EQ-5D (β = 0.04), EQ-VAS (β = 0.26), HLS-SF12 (β = 0.12), PSSS (β = 0.14)], those with an age of over 60 years old (β = 0.03) were more willing to accept it than those under 30 years old. Furthermore, being married (β = 0.08) and having 3 or more properties (β = 0.02) were also associated with a higher willingness to accept. The male group with children (β = −0.04) showed a lower willingness to accept.

### Subgroup analysis of hospice care acceptance in urban and rural areas

**Figure 5:**
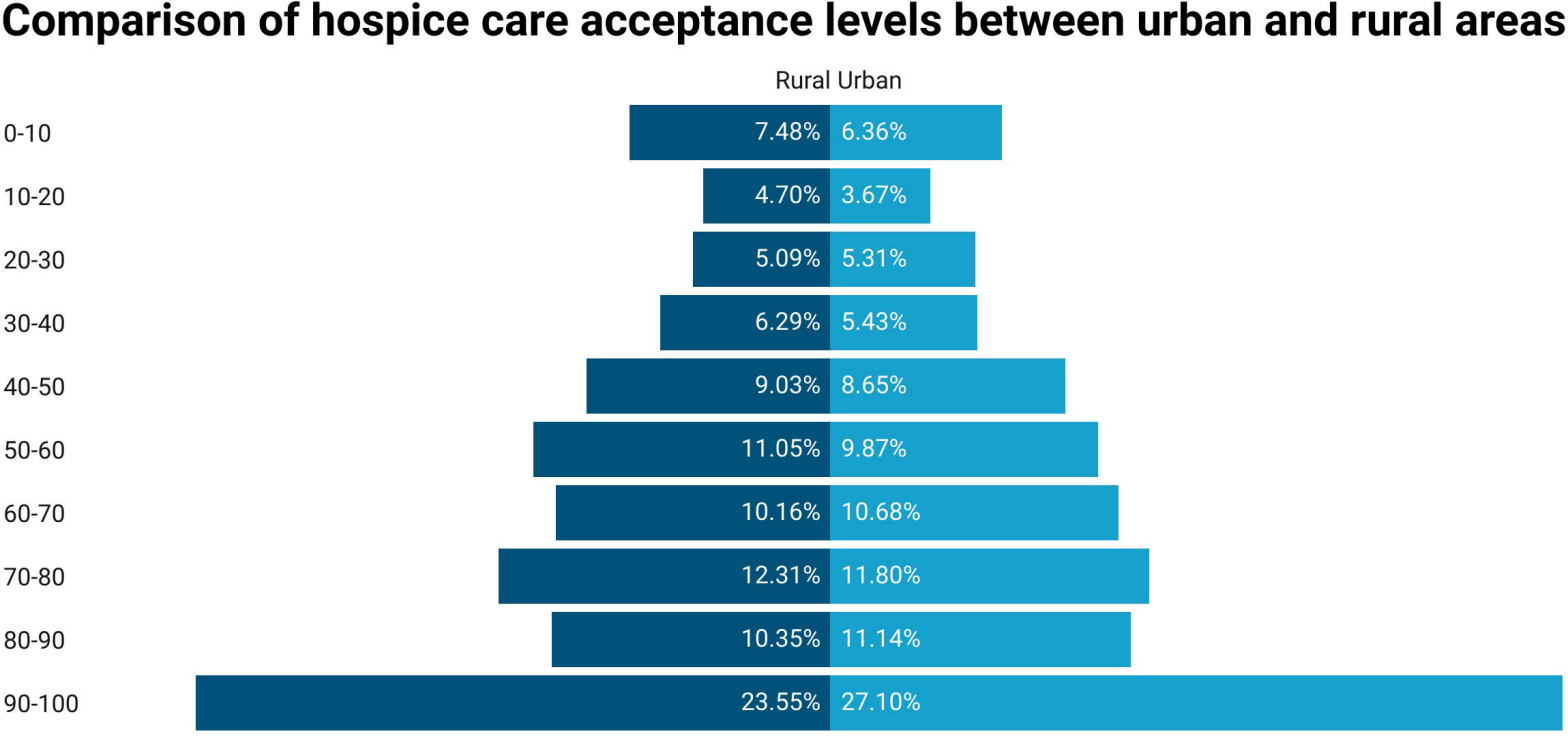
Comparison of hospice care acceptance levels between urban and rural areas.

Although the difference between urban and rural permanent residence was not significant in the multivariate regression results, the analysis of different groups of people in urban and rural areas may be conducive to the promotion of hospice care programs in urban and rural areas (Figure 5). For this reason, we further conducted a stepwise regression analysis of the urban and rural subgroups (Table 4). In the rural subgroup, the highest educational level [undergraduate or above (β = 0.04)], psychological status [mild anxiety (β = 0.03), moderate anxiety (β = 0.02), moderate stress (β = 0.08) and severe stress (β = 0.07)], and the four scales [SHMS (β = 0.08), EQ-VAS (β = 0.23), HLS-SF12 (β = 0.30), and PSSS (β = 0.11)] were consistent with the overall situation. Those with the age of 30–44 (β = 0.02) or 60 years old and above (β = 0.04), 3 or more properties (β = 0.02), and those diagnosed with chronic diseases (β = 0.02) showed a higher willingness to accept.

**Table 4:** Hierarchical stepwise regression analysis of predictors of hospice acceptance by place of residence.

**Variables**		**Unstandardized**		**Standardized**	***t***	***P***	**95%** ***CI***	
		**coefficients**		**coefficients**				
		***B***	***SE***	***Beta***			**Lower limit**	**Upper limit**
**Rural**								
Age group (Ref: <30)								
	30–44	3.27	1.48	0.02	2.20	0.028	0.36	6.18
	≥60	6.07	1.94	0.04	3.14	0.002	2.28	9.87
Highest educational level (Ref: Junior high or below)								
	Undergraduate or above	5.20	1.54	0.04	3.37	<0.001	2.18	8.23
Number of propertes owned (Ref :0)								
	≥3	8.85	3.14	0.02	2.82	0.005	2.69	15.01
Whether having chronic disease (Ref: No)								
	YES	3.79	1.61	0.02	2.35	0.019	0.62	6.95
Anxiety (Ref: No anxiety)								
	Mild anxiety	3.12	1.27	0.03	2.47	0.014	0.64	5.60
	Moderate anxiety	4.73	1.91	0.02	2.47	0.013	0.98	8.48
Pressure (Ref: Mild pressure)								
	Moderate pressure	6.44	1.29	0.08	5.01	<0.001	3.92	8.96
	Severe pressure	20.22	2.52	0.07	8.02	<0.001	15.28	25.16
SHMS		0.20	0.06	0.08	3.08	0.002	0.07	0.32
EQ–VAS		0.19	0.03	0.23	6.59	<0.001	0.14	0.25
HLS–SF12		0.58	0.09	0.30	6.49	<0.001	0.40	0.75
PSSS		0.16	0.05	0.11	3.50	<0.001	0.07	0.25
**Urban**								
Gender(Ref: Female)								
	Male	−2.71	0.65	−0.03	−4.17	<0.001	−3.98	−1.43
Highest educational level (Ref: Junior high or below)								
	Senior high or specialty	2.73	0.98	0.02	2.77	0.006	0.80	4.66
	Undergraduate or above	5.56	0.94	0.06	5.93	<0.001	3.72	7.41
Type of medical insurance (Ref: Self–pay and resident insurance)								
	Employee health insurance and commercial health insurance	4.76	1.95	0.04	2.44	0.015	0.94	8.57
	Government insruance	4.48	1.88	0.05	2.38	0.017	0.79	8.17
Anxiety (Ref: No anxiety)								
	Mild anxiety	4.00	0.78	0.03	5.11	<0.001	2.47	5.53
	Moderate anxiety	2.71	1.23	0.01	2.20	0.028	0.30	5.12
Pressure (Ref: Mild pressure)								
	Moderate pressure	3.79	0.80	0.04	4.76	<0.001	2.23	5.36
	Severe pressure	17.09	1.58	0.06	10.84	<0.001	13.99	20.18
SHMS		0.33	0.04	0.13	8.83	<0.001	0.25	0.40
EQ−5D		0.61	0.18	0.05	3.32	<0.001	0.25	0.97
EQ–VAS		0.17	0.02	0.20	8.99	<0.001	0.14	0.21
FHS–SF		0.30	0.06	0.16	4.79	<0.001	0.17	0.42
HLS–SF12		0.24	0.06	0.12	3.85	<0.001	0.12	0.36
PSSS		0.14	0.03	0.10	4.28	<0.001	0.08	0.21

In the urban subgroup, gender [male (β = −0.03)], highest education level [senior high or specialty (β = 0.02), undergraduate or above (β = 0.06)], medical insurance type [employee health insurance and commercial health (β = 0.04), government insurance (β = 0.05)], psychological state [mild anxiety (β = 0.03), moderate anxiety (β = 0.01), moderate stress (β = 0.04) and severe stress (β = 0.06)], and six scales [SHMS (β = 0.13), EQ-5D (β = 0.05), EQ-VAS (β = 0.20), FHS-SF (β = 0.16), HLS-SF12 (β = 0.12), and PSSS (β = 0.10)] also showed correlations consistent with the general trend.

## Discussion

This study is the first to examine the acceptance level of Chinese residents for hospice care and its influencing factors. The average score of residents in this study was 65.02, which is relatively high. It was found that most residents intended to receive hospice care. In addition, the study identified some significant factors that may influence attitudes, providing a reference for targeted hospice care promotion strategies.

Firstly, gender is a factor that influences the attitude of residents. Our study discovered that women were more willing to accept hospice care. This result was consistent with the cross-sectional study about receptivity to palliative care in New York (22), while other studies have found no association between gender and hospice care (23). Secondly, our research indicated that educational level is the critical factor in hospice care. The higher education people receive, the greater the acceptance of hospice care they have (24). This result was similar to that of a previous study, which reported that patients with an educational level of a college degree or above are evidently more willing to accept hospice care (25). Thirdly, when compared to residents who did not own a house property, those who owned two or more house properties expressed greater concerns for hospice care. In most regions of China, house property equals economic status, which is positively associated with people's health literacy. Meanwhile, our research also found that health literacy plays a significant role in the population's attitude toward hospice care. Residents with higher HLS-SF12, EQ-5D, and EQ-VAS scores were more likely to develop an intention, indicating that higher health attention is positively related to hospice care. Therefore, it was deduced that better economic conditions and higher levels of knowledge are positively associated with hospice care appeals.

Furthermore, when we analyzed the data by gender, we discovered some other intriguing predictors of hospice acceptance. Compared to women, age, marital status and house properties showed a positive correlation with hospice care in men. The factor of whether having children was just the opposite. These results specifically revealed differences in health and thanatopsis between men and women in China. Previous research has reported that men have higher occupational risks and lower health literacy than women (26). Therefore, when Chinese men's marriage demands, economic demands, and child-rearing demands are satisfied, they will give more priority to their demands for hospice care. What's more, better family atmosphere will help people choose palliative care when dying in the intensive care unit (27). In our research, the FHS-SF score was an important factor influencing women's choice of hospice care but not for men. It indicated that family health is essential for Chinese women when choosing hospice care.

As a traditional agricultural country, the exploration of urban-rural differences in hospice acceptance is also of great significance. The FHS-SF score was found to be a specific factor in urban areas, which may be due to more family-centered care in urban areas (28). Rural residents have limited health literacy because of some structural barriers like a shortage of specialist doctors, which leads to their lower scores for hospice care (29). As a result, younger people with relatively higher health literacy show higher scores for hospice acceptance than the aged (30). However, in the rural areas of China, older residents were more willing to accept hospice care when dying. This may not mean they are more health-literate than the young, but rather that they would prefer to die with a lighter burden on their children.

Notably, our results may provide helpful advice for healthcare providers to know more about patients' concerns. In our research, people with higher EQ-5D, FHS-SF and PSSS scores had a more acceptable intention toward hospice care. It was revealed that individual life support and emotional companionship are considered major factors in a good death (31). Another research found that higher PSSS scores mean higher psychological mediating ability, which may be the reason for accepting hospice care before death. Hospice care, as we know, involves providing people with adequate social support at the end of their lives. Most patients with cancer had supportive attitudes toward hospice care in a cross-sectional study in Mainland China. They wanted to know their diagnosis or prognosis of the disease, and wished to improve their life quality rather than prolong their life expectancy (32). In the countries with developed specialist hospice care, factors affecting people' preference are summarized as follows: available services, notably community support and hospice care units (33). Therefore, enhancing positive individual and social support may help alleviate the pain of dying people.

At the same time, our study reported that residents with high SHMS scores had a higher willingness to receive hospice care. Similarly, residents with higher anxiety and pressure levels were also more likely to accept hospice care. These people didn't have a greater health status, but showed a more urgent willingness for hospice care. The hypothesis of health behavior theory also showed that residents who are opposed to their own health risks are more likely to seek medical help (34). Pain caused by chronic disease, anxiety, and depression are other key components of palliative care (35). So reducing negative individual pains and social burden may prove to be necessary for clinicians.

One of our study's strengths is the large sample size, which slashes the influence of existing bias. It is the first time to discuss the public attitudes and explore the related factors on hospice care among a nationwide sample of the Chinese population. The perspective of this study can provide some valuable insight and reference for future research on hospice care policy theory. It can help researchers understand the process of accepting hospice care promotion better.

Despite some significant outcomes in hospice care, there are also limitations. Firstly, reporting bias was possible because of the self-reported information and the self-assessed scales in the study. Moreover, we could not know the number of participants who reviewed the online poster or survey but decided not to complete the survey, and thus could not assess non-response bias. Secondly, it is difficult to establish causality among the variables for the cross-sectional design of this study (27). Future research could explore more longitudinal studies to evaluate the relationship between various influencing factors and the acceptance of hospice care among residents. Besides, the distribution of the participants was imbalanced across the regions which might not be representative of the population. In addition, disparities in medical policy among different regions may exert an influential effect when people choose hospice care during their last days of life (36). More local and deep research a waits our exploration in the future.

## Conclusion

The study found that Chinese residents' acceptance of hospice care scored 65.02 points. Gender, house properties, educational level, anxiety, pressure, health care type, health literacy and social support were the main influencing factors of residents' attitudes. Governments and medical institutions should take targeted measures to increase social support and improve the mental health of the population. This can facilitate the popularization of death education and the implementation of hospice policies in hospitals.

## Data availability statement

The original contributions presented in the study are included in the article/supplementary material, further inquiries can be directed to the corresponding authors.

## Ethics statement

The studies involving human participants were reviewed and approved by the Institutional Review Committee of Jinan University, Guangzhou, China (JNUKY-2021-018). The patients/participants provided their written informed consent to participate in this study.

## Author contributions

XinZ, XunZ, YL, TC, YinW, and XY contributed to the conceptualization and methodology. YibW, YuW, XS, and W-KM contributed to investigation. YL, TC, and XY contributed to the design of the study and the data analysis. XinZ, XunZ, YL, TC, LS, YinW, and XY contributed to the writing of the manuscript. JW, YibW, and ZX critically revised the manuscript. All authors contributed to the article and approved the submitted version.

## Funding

This research was supported by the Youth Project of Key Research Bases of Philosophy and Social Sciences in the Sichuan Province (grant YF22-Q13).

## Conflict of interest

The authors declare that the research was conducted in the absence of any commercial or financial relationships that could be construed as a potential conflict of interest.

## Publisher's note

All claims expressed in this article are solely those of the authors and do not necessarily represent those of their affiliated organizations, or those of the publisher, the editors and the reviewers. Any product that may be evaluated in this article, or claim that may be made by its manufacturer, is not guaranteed or endorsed by the publisher.
